# Disparity in the Prevalence of Pit and Fissure Sealants Among 12-Year-Old Children in Puerto Rico: A Cross-Sectional Study

**DOI:** 10.21203/rs.3.rs-11053173/v1

**Published:** 2026-10-19

**Authors:** Milagros J. Toro, Augusto Elias-Boneta, Heriberto Gómez-Hierro, Juliana Iral, Margarita Murillo, Bayardo Garcia-Godoy, Sona Rivas-Tumanyan

## Abstract

Background Pit and fissure sealants (PFS) are an important tool in preventing and managing caries development on primary and permanent posterior teeth in children and adolescents. Among children living in Puerto Rico, particularly those of low socioeconomic status, this preventive measure has historically been underutilized. The completion of the Government Health Insurance (GHI) reform in 2000 marked a pivotal moment, expanding access to PFS across the entire island. In this study, we compared oral health data from 12-year-old school-attending children in Puerto Rico in 1997 and 2011 to evaluate changes in PFS prevalence and socioeconomic disparities, using school type as a proxy for socioeconomic status. Methods This cross-sectional study used two probabilistic samples (n = 1,587 in 2011 and n = 1,435 in 1997) of 12-year-olds enrolled in school, stratified by GHI region, school type, and sex. Calibrated examiners evaluated the presence of PFS on at least one permanent first molar. Logistic regression models compared PFS prevalence by school type in 1997 and 2011, adjusting for sex and region. A *P* -value ≤ 0.05 was considered statistically significant. Results Sex distribution and school type were similar in 1997 (49% female; 18% private schools) and 2011 (53% female; 23% private schools). Between 1997 and 2011, PFS prevalence increased among all children (4.3% vs. 18.1%), as well as among children attending private (11.1% vs. 25.8%), public rural (3.4% vs. 16.3%), and public urban schools (2.5% vs. 16.9%). Participants attending public-rural and public-urban schools were less likely to have sealants, both in 1997 ( *OR*_public−rural_ =0.19; 95% CI: 0.07–0.46; *OR*_public−urban_ =0.17; 95% CI: 0.06–0.48) and in 2011 ( *OR*_public−rural_ =0.57; 95% CI: 0.33–0.99; *OR*_public−urban_ =0.60; 95% CI: 0.39–0.93), versus those from private schools. Conclusions PFS prevalence increased among 12-year-old school-attending children in Puerto Rico across all school types between 1997 and 2011, a period that coincided with island-wide GHI implementation. However, disparities between children attending public and private schools persisted. These findings suggest that expanded insurance coverage alone may be insufficient to eliminate disparities in preventive dental care and that socioeconomic background may influence PFS utilization.

